# Dual Inhibition of Plasminogen Kringle 5 on Angiogenesis and Chemotaxis Suppresses Tumor Metastasis by Targeting HIF-1α Pathway

**DOI:** 10.1371/journal.pone.0053152

**Published:** 2012-12-31

**Authors:** Wei-Bin Cai, Yang Zhang, Rui Cheng, Zheng Wang, Shu-Huan Fang, Zu-Min Xu, Xia Yang, Zhong-Han Yang, Jian-Xing Ma, Chun-Kui Shao, Guo-Quan Gao

**Affiliations:** 1 Department of Biochemistry, Zhongshan School of Medicine, Sun Yat-sen University, Guangzhou, China; 2 DME Center, Clinical Pharmacology Institute, Guangzhou University of Chinese Medicine, Guangzhou, China; 3 Key Laboratory of Functional Molecules from Marine Microorganisms (Sun Yat-sen University), Department of Education of Guangdong Province, Guangzhou, China; 4 Department of Physiology, University of Oklahoma Health Sciences Center, Oklahoma City, Oklahoma, United States of America; 5 Department of Pathology, the Third Affiliated Hospital, Sun Yat-sen University, Guangzhou, China; 6 China Key Laboratory of Tropical Disease Control (Sun Yat-sen University), Ministry of Education, Guangzhou, China; Cedars-Sinai Medical Center, United States of America

## Abstract

We had demonstrated that plasminogen kringle 5 (K5), a potent angiogenic inhibitor, inhibited retinal neovascularization and hepatocellular carcinoma growth by anti-angiogenesis. The current study investigated the effects and the underlying mechanisms of K5 on both tumor growth and spontaneous pulmonary metastasis in Lewis lung carcinoma (LLC) implanted mouse model. Similarly, K5 could decrease expression of VEGF in LLC cells and grafted tissues and suppress tumor angiogenesis and growth. K5 had no direct effect on proliferation and apoptosis of LLC. However, K5 could significantly inhibit SDF-1α-induced chemotaxis movement of LLC cells and resulted in a great reduction of surface metastatic nodules and micrometastases in the lungs of LLC tumor-bearing mice. K5 also decreased expression of chemokine (C-X-C motif) receptor 4 (CXCR4) in LLC cells and grafted tissues. Furthermore, K5 down-regulated SDF-1α expression in metastatic lung tissues of LLC-bearing mice. Therefore, K5 may suppress tumor pulmonary metastasis through inhibiting SDF-1α-CXCR4 chemotaxis movement and down-regulation of VEGF. Moreover, the role of hypoxia inducible factor-1α (HIF-1α), a crucial transcriptional factor for both VEGF and CXCR4 expression, was evaluated. The siRNA of HIF-1α attenuated expression of VEGF and CXCR4 and inhibited LLC migration. K5 decreased HIF-1α protein level and impaired nuclear HIF-1α accumulation. These results showed for the first time that K5 inhibits LLC growth and metastasis via the dual effects of anti-angiogenesis and suppression of tumor cell motility by targeting the pivotal molecule, HIF-1α.

## Introduction

Tumor angiogenesis is a prerequisite for malignantly transformed cells to grow and metastasize and constitutes an important point in the control of cancer progression [1]. Hypoxia, a common feature of tissue microenvironments in both experimental and human solid tumors, induces or increases the gene expression of some factors that contribute to tumor angiogenesis and metastatic progression [2]–[4]. The hypoxic response is mediated through the transcription factors, hypoxia-inducible factor 1α (HIF-1α) [4]. Both clinical and experimental studies have revealed a significant association between the expression of HIF-1α and development and prognosis of malignant tumors [5]. HIF-1α signal pathways leading to the tumor angiogenesis and invasive migration have been suggested to be key steps of tumor metastatic progression [2], [4]. Thus, it was hypothesized that inhibition of HIF-1α signal pathway would therefore offer an innovative strategy for anti-angiogenesis and anti-metastasis in cancer therapy [3].

K5, the fifth kringle domain distinct from angiostatin in human plasminogen, has been shown to act as a potent inhibitor of endothelial cell and angiogenesis [6]. Previous researches demonstrated the anti-angiogenesis and anti-tumor activities of K5 or modified K5 in both the animal models of ischemia-induced neovascularization and grafted tumor, indicating that K5 may hold considerable potential in the treatment of neovascular diseases and solid tumor [7]–[13]. Recently, some studies focused on the effects of K5 on cell motility such as recruitment of tumor-associated macrophages [14], endothelial cell migration [15], aggregation of inflammatory cells [9] and neutrophil infiltration [16], suggesting a significant association between the biological activity of K5 and the regulation of cell motility in endotheliocytes and other cells. Chemotaxis and migration of tumor cells, the key steps in cancer metastasis, are induced and driven by hypoxia and closely related to HIF-1α signal pathway [17]. Lately, some studies show that VEGF secreted by primary tumor and chemokine (C-X-C motif) receptor 4 (CXCR4) expressed on the membrane of malignant cells play important roles in the process of metastasis [18], [19]. Both promoters of VEGF and CXCR4 genes contain hypoxia response element (HRE),which is the specific binding site for HIF-1α [20], [21].

The exact mechanism for anti-angiogenic and anti-tumor activity of K5 remains to be elucidated. Our previous study showed that down-regulation of HIF-1α by K5 is responsible for the decreased VEGF expression in endothelial cells [10], [22]. Based on these results, we hypothesize that K5 might inhibit cancer progression such as growth and metastasis by the dual effects of anti-angiogenesis and suppression of tumor cell motility, and involving in the regulation of HIF-1α pathway. To test this hypothesis, the present study was designed to investigate the effects and the underlying molecular mechanism of K5 on the growth and metastasis of LLC.

## Materials and Methods

### Cell Culture and Hypoxic Treatment

Malignant cell lines in this study include human lung adenocarcinoma epithelial cell line A549 and mouse Lewis lung carcinoma (LLC) were obtained from American Type Culture Collection (USA). The cells were maintained in Dulbecco modified eagle medium (DMEM, Gibco BRL, Gaithersburg, MD) supplemented with 10% fetal bovine serum (FBS, Gibco BRL, Gaithersburg, MD) and incubated at 37°C in a humidified atmosphere of 5% CO_2_. Mouse LLC cells were routinely maintained in C57BL/6 mice by subcutaneous transfer every 3 to 4 weeks. 4- to 6-week-old C57BL/6J mice were from Laboratory Animal Center, SUN Yat-sen University (Guangzhou, China). For hypoxic exposure in some experiments, the cells were cultured in the atmosphere with 0.5% oxygen, 5% CO_2_, and 94.5% nitrogen at 37°C for indicated hours.

### Animal Experiments and Histology Analysis

1×10^6 ^LLC cells were inoculated subcutaneously into the dorsal area of C57BL/6J mice. The mice bearing LLC tumor were randomized into two groups on the 7^th^ day following the inoculation and received intraperitoneal injection of K5 or PBS, respectively. Human K5 was expressed, purified, and analyzed as described previously [7], [12]. The K5 group received the injection of K5 at the dosage of 2.5 mg/kg per mouse every three days, until the overall dosage reached 10 mg/kg. The control group was treated with the same volume of PBS. The mice were closely monitored and body weight and tumor volume were recorded every two days. The relative mouse body weight was calculated by the ratio of the measured body weight to the initial and tumor volume was determined by the formula: volume = length×width^2^×0.5. At the termination of experiments (22 days following the tumor cell injection), tumor tissues were collected and weighed.

The experimental animal model of spontaneous pulmonary metastasis was established as described by O'Reilly with some modification [23]. Briefly, after 7 days of LLC cell inoculation, the subcutaneous primary tumor was removed by aseptic operation under chloral hydrate anaesthesia. Then the animals were divided into two groups and treated with K5 as stated above. Mice were sacrificed 6 days after the last injection of K5, and their lungs were fixed overnight in 4% neutral buffered paraformaldehyde for pathologic analysis. The metastatic nodules at the lung surface and pulmonary micrometastases were counted by macroscopic and stereomicroscope observation, respectively. The alveolar cross-sectional area was calculated as previously described to indicate the pulmonary micrometastases and inflammatory reaction. Alveolar spaces were captured electronically and total alveolar area was obtained from the sum of individual alveolar air spaces within the whole lung region of each slide. The area of the lung (Vp) and the area of the alveoli (Va) were definite and calculated. The proportion of alveoli to lung tissue was presented by Va/Vp.

### Ethics Statement

The study was approved by the Animal Care and Ethics Committee of SUN Yat-sen University, and all animal studies were performed under an institutionally approved protocol according to the guidelines and the criteria from the committee. All surgery was performed under chloral hydrate anaesthesia, and all efforts were made to minimize suffering.

### Microvascular Density Assay

The tissues were fixed in 4% paraformaldehyde solution and prepared as paraffin-embedded samples for immunohistochemical analysis. The endothelial cells of tumor samples were identified by immunostaining with the primary antibody for mouse CD34 (1∶100 Santa Cruz Biotechnology) at 4°C overnight. The avidin-biotin-peroxidase technique was used for detection of immunolabeled cells and the sections were counterstained with hematoxylin. In negative-control staining, the primary antibodies were omitted. Tumor microvessel density was quantified as tumor vasculature by the Weidner’s method [24].

### Western Blot Analysis

Western blot analysis was performed as described in detail previously [25]. Briefly, after separation in polyacrylamide gel, the proteins were transferred to PVDF membrane and subjected to Western blot analysis with polyclonal antibodies against VEGF (Santa Cruz, diluted 1∶1000), CXCR4 (BD Biosciences, diluted 1∶1000), SDF-1α (Santa Cruz, diluted 1∶1000), HIF-1α (BD Biosciences, diluted 1∶1000) at 4°C overnight. Then the membranes were incubated with horseradish peroxidase-conjugated secondary antibodies, and visualized using an enhanced chemiluminescence detection kit (Pierce Biotechnology, Rockford, IL) according to the manufacturer’s recommendations. The β-actin (Sigma, diluted 1∶10000) or Histone 2B (Santa Cruz, diluted 1∶2000) was applied as control.

### Immunofluorescence and Immunocytochemistry

Direct immunofluorescence was applied to determine the expression of CXCR4 on the membrane of LLC cells. The cells were seeded in 6-well plates with gelatin-coated coverslip at a density of 2×10^5^ cells per well. When grown to 80% confluency, cells were starved with serum-free DMEM overnight and then incubated with K5 at different concentrations in normoxia or hypoxia for 12 h. Cells were fixed in 4% paraformaldehyde solution, blocked in 3%BSA, and incubated with anti-CXCR4 monoclonal antibody labeled by phycoerythrin (R&D Systems) at 4°C overnight. After stained with propidium iodide (PI) for 2∼3 min, the images of green fluorescent (PE) and red fluorescent (PI) were captured and then transferred to an image analysis program (ImagePro Plus 6.0, Media Cybernetic) to merge together and analyze.

Immunocytochemistry was performed as described in detail previously [26]. Briefly, LLC cells were seeded in 6-well plates with gelatin-coated coverslip and treated with K5 for 12 h as mentioned above. The change of HIF-1α was detected by immunocytochemistry assay (anti-HIF-1α 1∶100, Santa Cruz Biotechnology).The images of HIF-1α were captured by an Olympus microscope at×400 magnification. ImagePro Plus 6.0 was applied to analyze the cellular localization of HIF-1α in LLC cells.

### siRNA Silencing of HIF-1α

Two HIF-1α shRNAs (hifA and hifB) targeting the 19-nucleotide 5′- GATGGAAGCACTAG ACAAA-3′ or 5′-CAAGCAACTGTCATATATA-3′ in the coding sequence region of human HIF-1α mRNA (NCBI accession number NM_010431) were designed using siRNA-designing software. The oligonucleotide pairs were annealed and ligated into vector pSilencer 1.0-U6 (Ambion, Austin, TX, USA) at A*pa*I and E*co*RI sites. The LLC cells were transfected with plasmids or empty vector by Lipofectamine 2000 (Invitrogen). After 6 h the transfection medium was changed to DMEM, and cells were exposed to hypoxia condition for indicated times. The silencing efficiency was verified by Western blot analysis.

### Chemotaxis

Cell chemotactic movement induced by SDF-1α was examined by a modified Boyden Chamber assay with 12-well chemotaxis chambers (Transwell, Corning). SDF-1α was added to the lower wells, and 5×10^4^ cells were added to each of the upper wells with or without K5 in different concentrations. The chemotaxis chambers were then incubated for 12 h at 37°C in normoxic or hypoxic condition. Cells were fixed in 4% polyoxymethylene solution and stained with hematoxylin and eosin or crystal violet. The number of cells, which had migrated through the membrane pores to the underside of the filters was calculated by counting the total number of cells in 5 separate fields of view under 400× magnifications.

### Cell Viability and Apoptosis Assays

LLC cells were seeded in 24-well plates in triplicate and cultured in the DMEM with 10% FBS medium to 60∼70% confluency. Cells were maintained in serum-free medium overnight, and then treated with different concentrations of K5 in DMEM without FBS for 48 h in 37°C, 5%CO_2_ condition. The number of viable cells at the end of the drug incubation was determined using a colorimetric MTT assay. Data from three independent tests represented absorbance as percentages of respective controls. For quantitative analysis of apoptosis, LLC cells were seeded in 6-well plates at a density of 2.5×10^4^ cells per well. After starved with serum-free DMEM overnight, cells were exposed to K5 at different concentrations for 48 h. Then the cells were harvested for Annexin and PI staining using the Annexin V-FITC Apoptosis Detection Kit (Sigma, St. Louis, Mo., USA). Cells treated with 10 µmol/L colchicine were used as positive control, and treated with PBS as negative control. The cells were subsequently counted by flow cytometry.

### Electrophoretic Mobility Shift Assay (EMSA)

LLC cells were cultured and treated as above. Nuclear extracts of LLC cells were collected using the kit from Activemotif (Tokyo, Japan) following the manufacturer’s instructions. The double-stranded oligonucleotides for HIF-1α binding (5`-GAGATAGCTGGGACCGAGGCG CGTGCGTCGCGACACGGACCCCAGAA-3) and a mutant DNA sequence (5` -GAGATAG CTGGGACCGAGGCGTTTTCGTCGCGACACGGACCCCAGAA-3) were used in EMSAs as probes and/or competitors. Both oligonucleotides were synthesized and chemically labeled with biotin at the 3` end. Nuclear proteins (5 µg) and 1 pmol probe were used in a 20-µl reaction using the binding buffer provided in the kit. Specific binding was controlled by competition with unlabeled probe (100 pmol) or mutant probe (100 pmol). For supershift assay, 2 µg antibody for HIF-1α (BD Biosciences) was added to the reactions. All DNA protein complexes were resolved by electrophoresis on 6% non-denaturing polyacrylamide gel and transferred onto a nylon membrane (Pierce Biotechnology). DNA was cross-linked to the membrane under UV light. The biotin end-labeled DNA was detected using the streptavidin–horseradish peroxide conjugate and LightShift chemiluminescent substrate (Pierce Biotechnology).

### Statistical Analysis

Experiments were routinely repeated usually three or more times and data were presented as means ± SD. Multiple group comparisons test was performed with one-way analysis of variance (ANOVA) by SPSS 11.0 software. A value of P<0.05 was considered statistically significant.

## Results

### Inhibitory Effects of K5 on Tumor Growth and Metastasis of LLC *in vivo*

**Figure 1:**
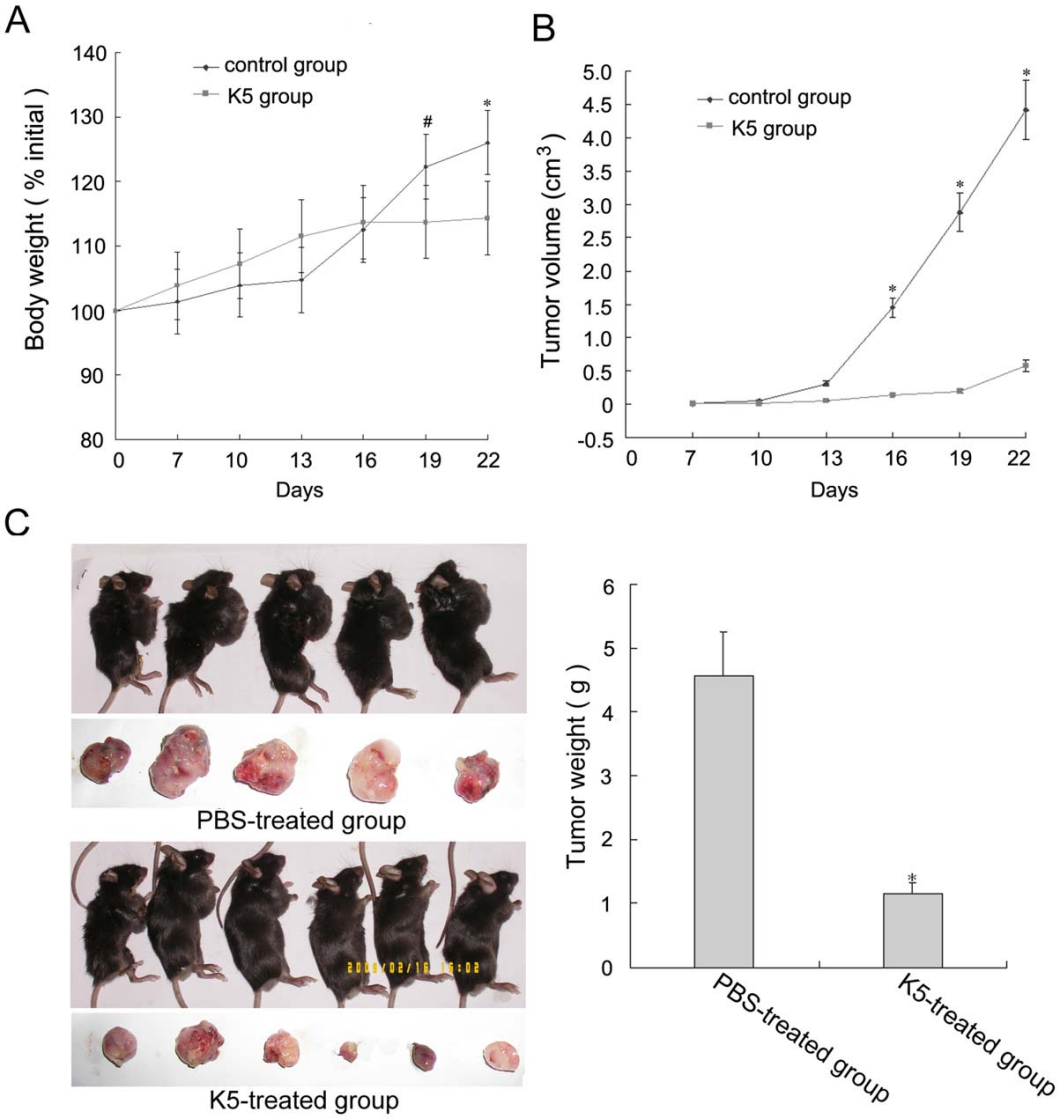
K5 inhibits tumor growth in LLC tumor-bearing mice. (A) Body weight curve of animals treated with K5 (▪) or PBS (⧫) on days indicated. (B) Tumor growth curves of LLC model with the treatment of K5 (▪) or PBS (⧫) and observation of tumor volume for 16 days after treatment. (C) Tumor tissues from the transplanted LLC mice model treated with K5 or PBS were collected (left) and the tumor weight was recorded (right). Data are presented as mean ± SD. Values significantly lower than control are indicated (^*^ *P*<0.01, ^#^ *P*<0.05).

To evaluate the suppressive effect of K5 on tumor growth, LLC-grafted model was established by implanting LLC cells into the dorsal area of C57BL/6 mice. From day 9 after K5 treatment until the end of the experiment, there was a marked failure to gain body weight in K5-treated mice relative to that of the control (Fig. 1A). Similarly, tumor growth rates of K5-treated group appeared lower as compared with the control group (Fig. 1B). At day 9 after K5 treatment initiation, the means of the tumor volumes were 1.45 cm^3^ and 0.139 cm^3^ for K5-treated group and PBS-treated group, respectively. As shown in figure 1C, the mean tumor weight in K5-treated group was remarkably lower than that in the PBS-treated control group, indicating the suppression of primary tumor growth (*p*<0.01, Fig. 1C). These data demonstrated that K5, with the dose of 10 mg/kg, significantly inhibited tumor growth in LLC-grafted model.

**Figure 2:**
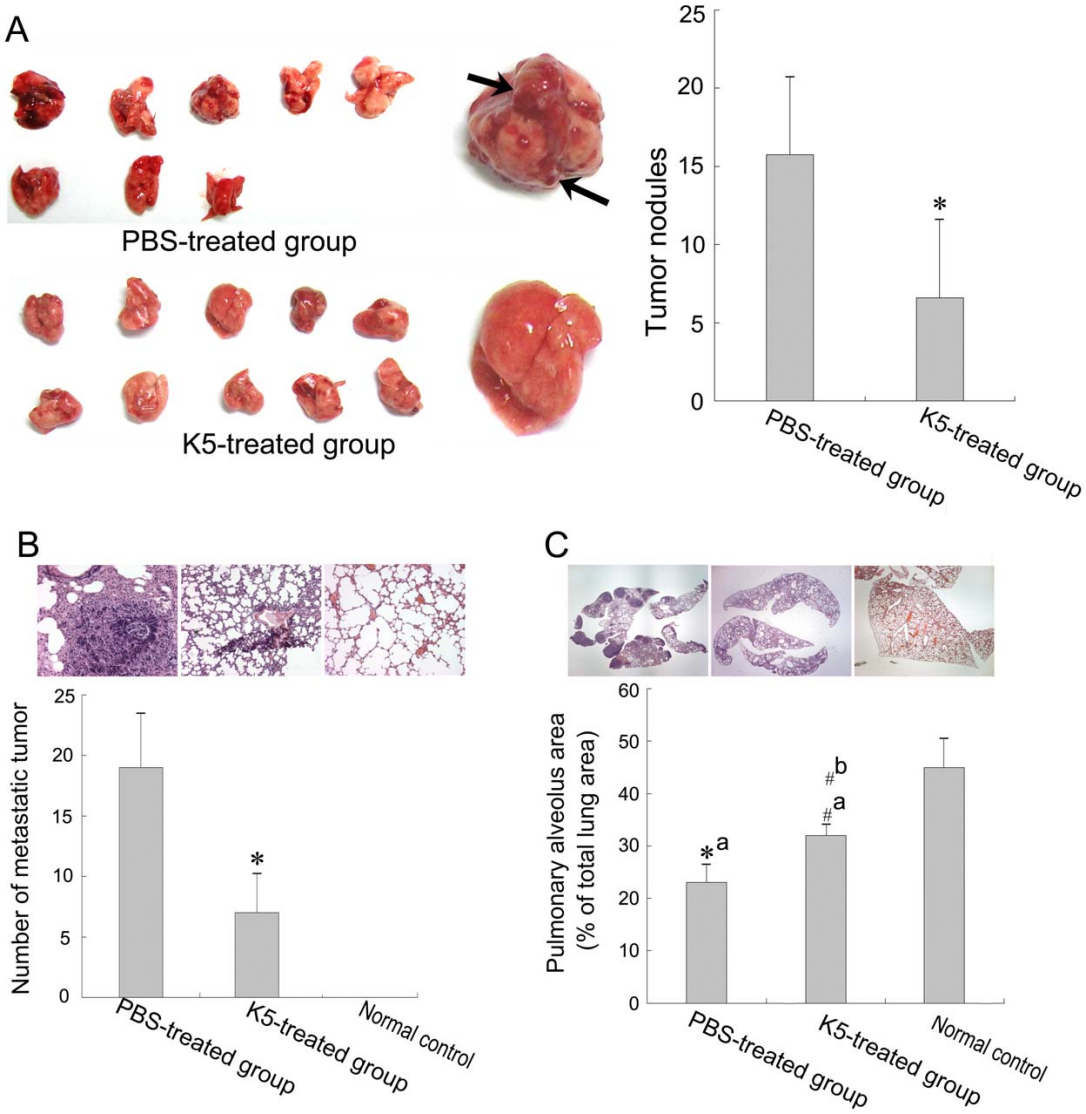
K5 suppresses pulmonary metastasis of LLC. (A) The surface nodules on mouse lung (left, marked by black arrow) and quantitative analysis (right, ^*^ *P*<0.01, *vs* PBS control group). (B) Micrometastases in lung observed with high power microscope (×200) and quantitative analysis (^*^ *P*<0.01, *vs* PBS control group). (C) Pulmonary alveoli observed with low power microscope (×20) and quantitative analysis of pulmonary alveoli area in lung (^*^ *P*<0.01, ^#^ *P*<0.05; a: *vs* normal control group, b: *vs* PBS control group).

We also established a spontaneous pulmonary metastasis mouse model of LLC to determine the inhibition of K5 on tumor metastasis. Mice were sacrificed on the 15^th^ day after K5 treatment initiation and histological sections of their lung were stained with hematoxylin and eosin. In the untreated group, over 80% of mice showed macroscopical metastasis nodules on pulmonary surface (Fig. 2A, marked by the black arrow) and all mice had low pulmonary alveoli area in comparison with the normal control. K5 treatment significantly reduced the pulmonary metastasis nodules (*p*<0.05, Fig. 2A) and improved the survival rate of mice (data no shown). At high magnification, it was observed that the number of pulmonary micrometastases decreased markedly (*p*<0.05, Fig. 2B) and the pulmonary alveoli relative area increased in the K5-treated lungs with morphological characteristic quite close to the normal lung when compared to the untreated group (*p*<0.05, Fig. 2C). These results suggested that K5 significantly suppressed the spontaneous pulmonary metastasis of LLC.

### K5 Suppressed Tumor Angiogenesis and Down-regulates VEGF Expression in LLC Cells

**Figure 3:**
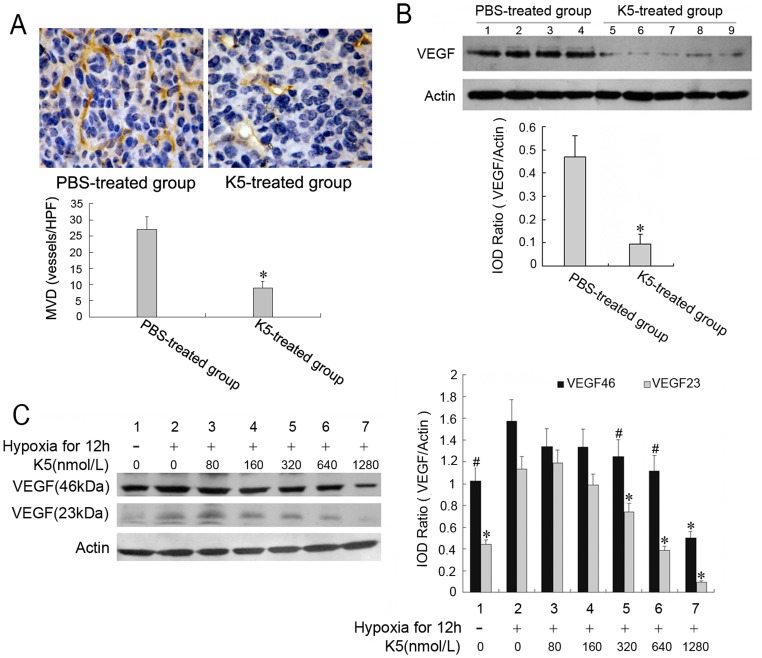
Inhibition of K5 on tumor angiogenesis and VEGF expression in LLC cells. (A) MVD in tumor tissues were determined by immunohistochemical staining (×400) and counted from six randomly selected fields every section (^*^ *P*<0.01, *vs* PBS control group). (B) Down-regulation of VEGF expression in tumor tissues from LLC grafted mouse model with K5 treatment. (C) Down-regulation of VEGF expression in LLC cells treated with K5. Changes in VEGF expression were determined by Western blotting analysis. Results are representative of three separate experiments. (^*^ *P*<0.01, ^#^ *P*<0.05; *vs* control group).

To determine whether the delay of tumor growth and metastasis in the mice with K5 treatment was related to angiogenesis, a careful examination of microvessel density (MVD) was performed by CD34 immunostaining for capillaries in tumor tissues. In animals that received intraperitoneal injection of K5, there was significant reduction of microvessel density in tumor tissues compared with the PBS control (Fig. 3A). As VEGF derived from tumor cells is the critical one of growth factors enhancing tumor microvessel density and inducing angiogenesis, we evaluated the effect of K5 on the expression of VEGF in LLC cells and tissues. As shown in figure 3B, K5 injection decreased VEGF expression to approximate 22.3% of the control in tumor tissues from the grafted LLC mouse model. This inhibitory effect was also examined in the cultured LLC cells. Consistent with the results in tumor tissues, K5 markedly reduced the level of cellular VEGF induced by hypoxia (Fig. 3C). These results suggested that the inhibitory effects of K5 on LLC tumor growth and angiogenesis may be through down-regulation of VEGF.

### K5 Inhibited Chemotaxis Movement but not Proliferation of LLC Cells

**Figure 4:**
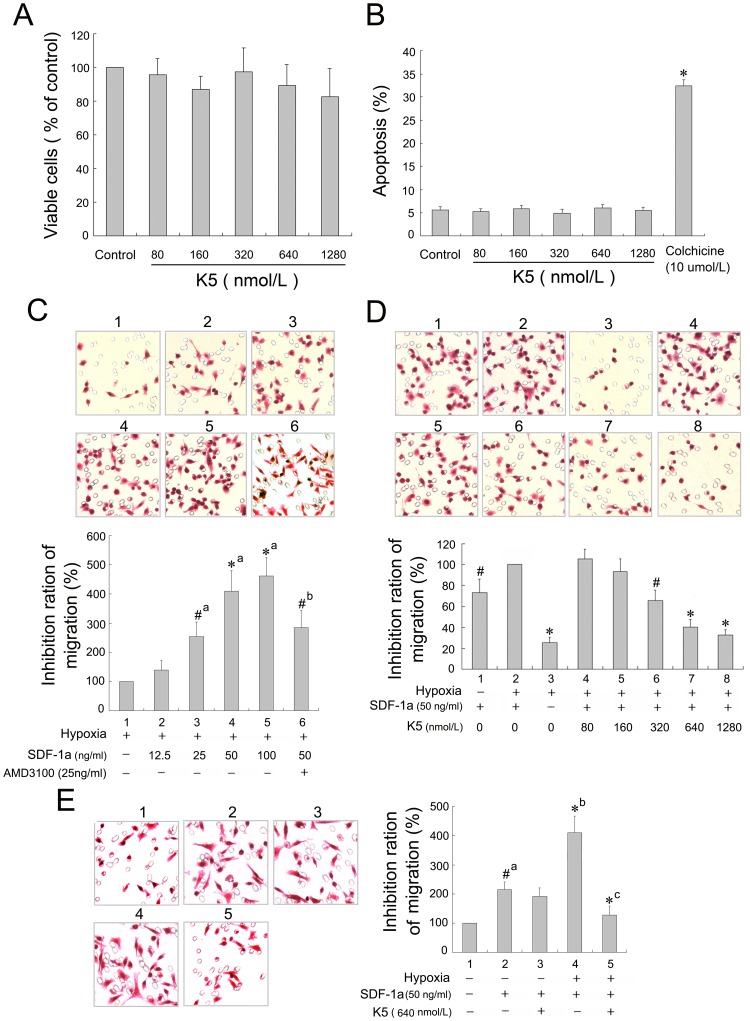
Effect of K5 on the proliferation, apoptosis and migration of LLC cells. (A) The viable cells were quantified by using the MTT assay and data presented absorbance as percentages of respective controls (means ± SD, *n* = 3). (B) Apoptotic cells, stained with Annexin V and propidium iodide, were quantified by flow cytometry with the negative control (PBS group) and the positive control (colchicines group) (means ± SD, *n* = 3, ^*^ *P*<0.01 *vs* control of group). (C), (D) & (E) The migrated cells were observed in a modified Boyden Chamber assay with HE staining (×400). Data are presented as a percentage of inhibition (*n* = 3. C,^ #^ *P*<0.05,^ *^ *P*<0.01, a: *vs* group 1, b: *vs* group 4. D, ^#^ *P*<0.05,^ *^ *P*<0.01 *vs* control group 2. E,^ #^ *P*<0.05,^ *^ *P*<0.01, a: *vs* group 1, b: *vs* group 3, c: *vs* group 4).

Our previous studies demonstrated that K5 suppressed angiogenesis by inhibiting proliferation and inducing apoptosis of vascular endothelial cells [7]–[12], [22]. In this experiment, we explored the direct effect of K5 on LLC cells *in vitro*. LLC cells were treated with K5 at different concentrations of 0, 80, 160, 320, 640 and 1280 nmol/L for 48 h. As shown in figure 4A and B, K5 had no apparent effect on proliferation and apoptosis of LLC cells (the cells treated with 10 µmol/L cholchicine as a positive control). Even at the highest concentration of 1280 nmol/L, there was no apparent inhibitory effect observed.

However, K5 suppressed the chemotaxis movement of LLC cells induced by SDF-1α (50 ng/ml) under hypoxia condition. SDF-1α and its specific receptor CXCR4, which expressed on the membrane of tumor cells, form the SDF-1α/CXCR4 chemokine axis and play a pivotal role in migration, invasion and metastasis of some malignant tumors, such as melanoma, breast cancer and non-small cell lung cancer cells [27], [28]. As shown in figure 4C & D, SDF-1α had an apparent role in inducing the chemotactic movement of LLC cells in a concentration-dependent manner. And the SDF-1α-induced chemotaxis was enhanced significantly in LLC cells with the hypoxia treatment (*p*<0.05). Administration of K5 (320∼1280 nmol/L) reduced the number of migrated LLC cells in hypoxia as compared with the control (*p*<0.01), exhibiting a concentration-dependent inhibitory effect on the cell chemotaxis movement induced by SDF-1α (Fig. 4D). But there was no obvious inhibitory effect of K5 on the cell movement of LLC under normoxia condition (Fig. 4E).These suggested that the anti-metastatic effect of K5 might be due in part to blocking chemotaxis movement of LLC cells induced by SDF-1α.

### Blocking CXCR4 Suppressed the Migration of LLC Cells

**Figure 5:**
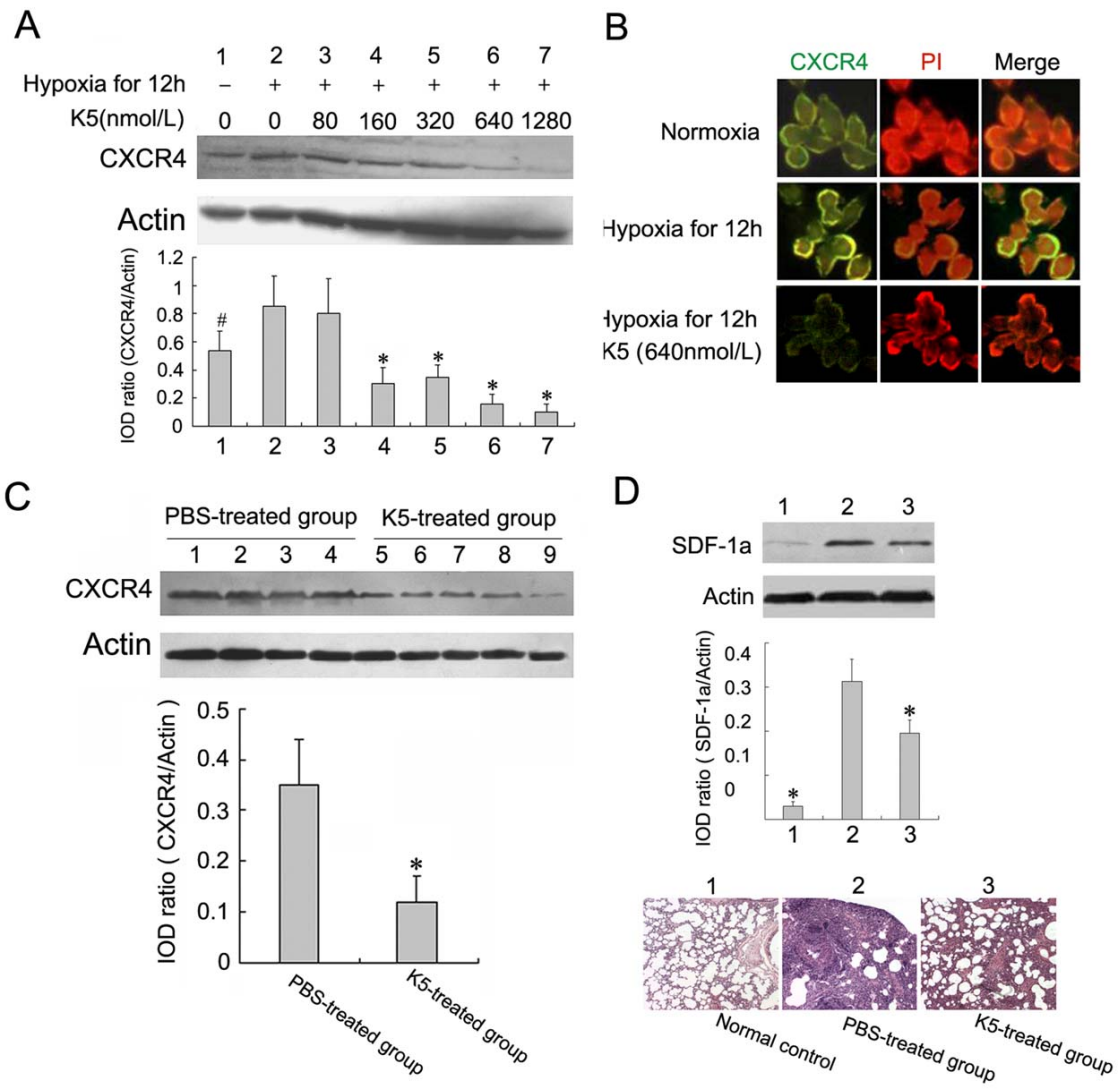
K5 down-regulates the expression of CXCR4 in LLC cells. (A) K5 dose-dependently down-regulated CXCR4 expression in tumor cells treated with hypoxia, and changes in CXCR4 expression were determined by Western blotting analysis (^#^ *P*<0.05, ^*^ *P*<0.01, *vs* PBS control group). (B) Membrane CXCR4 expression was decreased in LLC cells with K5 treatment evaluated by immunofluorescence assay (×640). (C) Down-regulation of CXCR4 expression in tumor tissues from LLC grafted mouse model with K5 treatment, and changes in CXCR4 expression were determined by Western blotting analysis (^*^ *P*<0.01, *vs* PBS control group). (D) K5 down-regulated SDF-1α in metastatic lung tissues of LLC by Western blotting analysis (^*^ *P*<0.01, *vs* PBS control group). The bottom HE-staining images represented morphological observation of lungs for the above three groups (×200).

**Figure 6:**
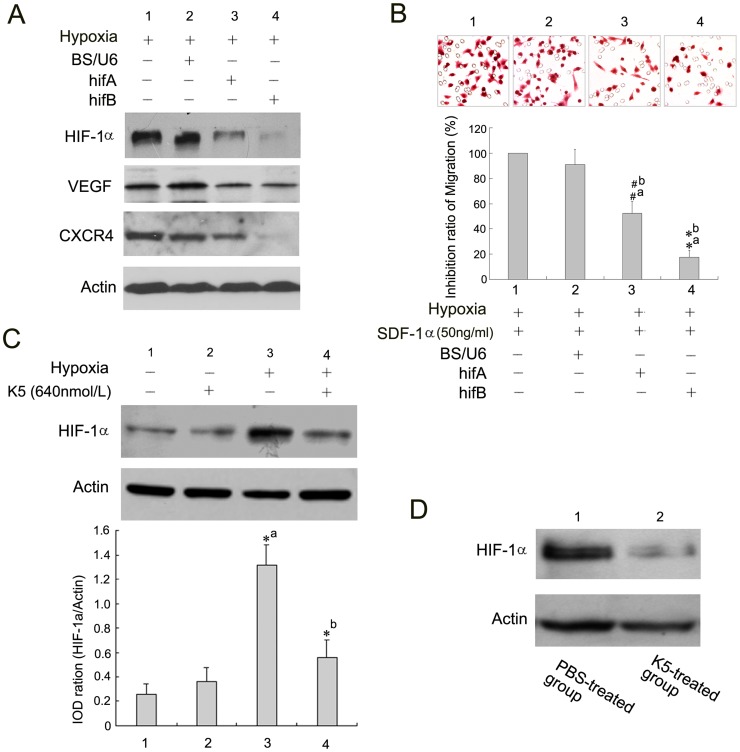
K5 suppresses the expression of CXCR4 and VEGF and LLC migration via HIF-1α inhibition. (A) Small interfering RNA targeting HIF-1α inhibits gene expression of CXCR4 and VEGF in LLC cells. (B) Cell migration detection after HIF-1α gene silencing (^#^ *P*<0.05,^ *^ *P*<0.01, a: *vs* group 1, b: *vs* group 2). (C) HIF-1α was down-regulated in LLC cells with K5 treatment by Western blotting analysis. Data are representative of three separate experiments (^*^ *P*<0.01, a: *vs* group 1, b: *vs* group 3). (D) Down-regulation of HIF-1α protein level in tumor tissues from LLC grafted mouse model with K5 treatment, and changes were determined by Western blotting analysis.

We next detected the expression of CXCR4 in LLC cells both *in vitro* and *in vivo* and evaluated the function of CXCR4 in the cell migration. Direct immunofluorescence was applied to determine the distribution of CXCR4 in LLC cells with anti-CXCR4 monoclonal antibody labeled by phycoerythrin, and the fluorescent microscope images showed that CXCR4 mainly distributed on the membrane of LLC cells as shown in figure 5B. The expression of CXCR4 on the cell surface was enhanced under hypoxic conditions compared to the normoxic controls in cultured LLC cells (Fig. 5A & B). Correspondingly, the western blotting analysis showed that the protein of CXCR4 expressed highly in tumor tissues from the grafted LLC mouse model (Fig. 5C). As CXCR4 is the only physiologic cognate receptor of SDF-1α, the evident concentration-dependent chemotactic responses to a gradient of SDF-1α (12.5∼100 ng/ml) revealed the expression and function of the chemokine receptor in LLC cells (Fig. 4C). To further define the roles of CXCR4 in cell migration, we determined whether AMD 3100, an antagonist binding to CXCR4, blocked the tumor cell movement induced by SDF-1α. As shown in figure 4C, 25 ng/ml AMD 3100 treatment significantly prevented cell migration towards SDF-1α in an *in vitro* system, and the migrated cells number of AMD-treated group decreased by about 40% compared with the control. In consideration of the regulation of hypoxia on the cell movement, we next evaluated the effects on the SDF-1α-induced cell migration by HIF-1α, a major nuclear transcription factor modulating gene expression in response to hypoxic conditions. We observed that the blocking agent of shRNA targeting HIF-1α suppressed SDF-1α-induced cell movement (Fig. 6B). The effectiveness of HIF-1α shRNAs was indentified by western-blot analysis as in Fig. 6A. These data suggested that CXCR4 was involved in the migration of the metastatic malignant cell and regulated by hypoxic conditions and HIF-1α in LLC cells.

### K5 Down-regulated the CXCR4 Expression and Inhibits the SDF-1α/CXCR4 System

For some malignant tumors, CXCR4 plays important roles in cell chemotaxis movement, which is essential for metastasis and inhibited by K5 as mentioned above. In this study, we determined whether K5 regulated the expression of CXCR4 in LLC cells and tissues. Western blotting analysis showed that K5 treatment markedly down-regulated CXCR4 expression in cultured LLC cells induced by hypoxia. Densitometric measurement of bands demonstrated CXCR4 protein levels in cultured LLC cells treated with K5 were reduced in a dose-dependent manner (Fig. 5A). We next examined the effect of K5 on the membrane CXCR4 expression in LLC cells by direct immunofluorescence staining mentioned above. Compared with the control group, the green fluorescence distributed on the membrane decreased significantly in LLC cells treated with 640 nmol/L K5 for 12 h, indicating the down-regulation of CXCR4 expression (Fig. 5B). This inhibitory effect was also examined in tumor tissues. Consistent with the results in cultured cells, K5 injection decreased CXCR4 protein level to approximate 45% of the control in LLC mouse model (Fig. 5C). Moreover, as shown in figure 5D, K5 can significantly down-regulate SDF-1α expression in metastatic lung tissues of LLC, showing the metastatic niche altered by K5 treatment. These results demonstrated that K5 inhibited the metastasis of LLC partially through the inhibition of SDF-1α/CXCR4 chemokine receptor system which involved in chemotaxis movement.

### K5 Suppressed the Expression of CXCR4 and VEGF and LLC Migration via HIF-1α Inhibition

**Figure 7:**
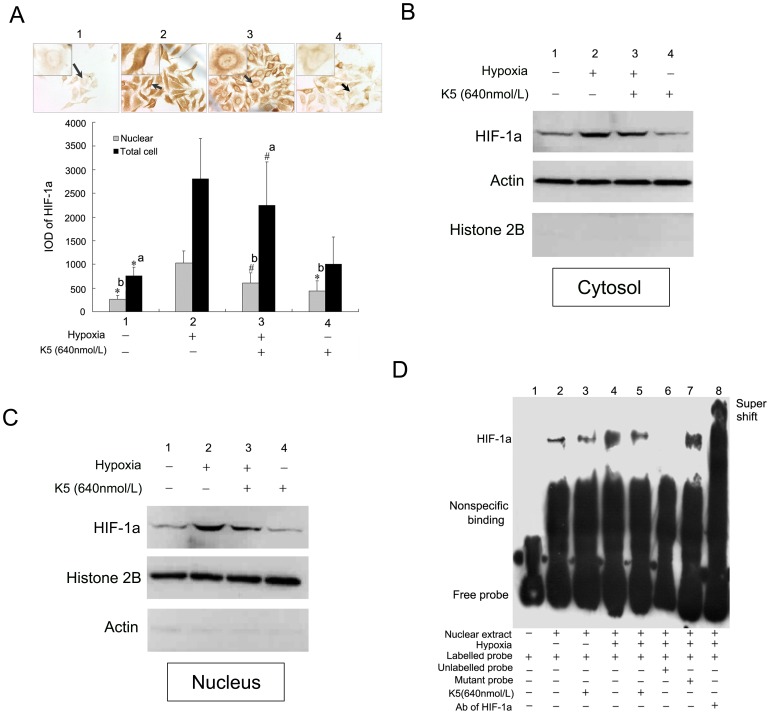
Effect of K5 on HIF-1α intracellular localization and transactivation in LLC cells. (A) Immunocytochemistry staining of HIF-1α in LLC cells and quantitative analysis with the software of Imagepro Plus 6.0 (×400). Data are representative of three separate experiments (^#^ *P*<0.05, ^*^ *P*<0.01, a: *vs* group 2 of whole cell, b: *vs* group 2 of nucleus).(B) and (C) Western blot analysis of cytoplasmic and nuclear fractions of LLC cells incubated under hypoxic conditions in the presence of K5. Actin served as cytoplasmic markers while histone 2B served as nuclear markers. (D) *In vitro* binding of nuclear extracts from LLC cells were assayed by gel shift using biotinylated probes of HRE. Lane 1, free probe control (without nuclear extracts). Lane 2–5, addition of nuclear extracts from K5-treated group or control group with hypoxia treatment or not. Competition reaction were performed using 100-fold molar excess of unlabeled wild-type probes (lanes 6), or mutant probe (lane 7), or super shift (lane 8).

Considering the regulative effect of hypoxic condition on the gene expression of VEGF and CXCR4 as described above, we hypothesized that the suppressive regulation of K5 on gene expression of VEGF and CXCR4 may be associated with HIF-1α. To test this hypothesis, two shRNAs (hifA and hifB) targeting HIF-1α were constructed with pSilencer 1.0-U6 vector for assessing the effects of HIF-1α on gene expression of VEGF and CXCR4. As shown in figure 6A, both shRNAs significantly inhibited the gene expression of HIF-1α in LLC ells. After transfection of the interference plasmids for 48 h, it was found that the expression of VEGF and CXCR4 in LLC cells were obviously inhibited in comparison with the control. Meanwhile, SDF-1α-induced cell movement of LLC was suppressed by small interfering RNA targeting HIF-1α (Fig. 6B). Then, we further evaluated the effect of K5 on the protein level of HIF-1α in LLC cells. K5 significantly down-regulated HIF-1α expression induced by hypoxia in the whole cells of LLC, which was confirmed by protein immunoblot test (Fig. 6C) and immunocytochemical staining (Fig. 7A). In the *in vivo* studies the inhibitory effect was also confirmed with tumor tissues. As shown in figure 6D, K5 injection greatly reduced the protein level in LLC tissues comparing with the control group. However, K5 has no effect on the expression of HIF-1α protein under the normoxic conditions (Fig. 6C & 7A). These suggested that HIF-1α inhibition might involve in the down-regulative effect of K5 on the expression of VEGF and CXCR4 in LLC cells.

### K5 Reduced Nuclear HIF-1α Accumulation and Impaired HIF-1α DNA-binding and Transactivation Function

We then investigated the effect of K5 on intracellular localization of HIF-1α. In normoxia, HIF-1α could hardly be detected, while a strong signal of nuclear HIF-1α accumulation was visible in LLC cells under hypoxic conditions (Fig. 7A). However, the nuclear signal became weaker in LLC cells with the treatment of K5, and HIF-1α was redistributed in both nucleus and cytoplasm. To confirm this phenomenon, the subcellular distribution of HIF-1α was also investigated in LLC cells by protein immunoblot, which was monitored using actin as cytoplasmic markers while histone 2B as nuclear markers. The significant up-regulation of HIF-1α in both cytoplasm and nuclear fraction was detected in LLC cells under hypoxic conditions (Fig. 7B). Treatment with K5 resulted in the reduction of HIF-1α levels, especially in nuclear (Fig. 7B & C).

To investigate the influence of K5 treatment on the formation of the HIF-1α protein–DNA complex, EMSA assays were performed using nuclear extracts from LLC cells. We prepared oligonucleotide probe covering 5′-GCGTG-3′ box, which was the core sequence of HRE. The specificity of the complex was suggested by the competition assays. As shown in figure 7D lane 6 and 7, an unlabeled probe completely blocked the formation of protein–DNA complex while oligonucleotides mutated in 5′-GCGTG-3′ box had no effect, confirming the specificity of the complex for HRE element. The identity of the specifically shifted protein was further confirmed using the antibody directed against HIF-1α (Fig. 7D lane 8). We further examined the ability of K5 to interfere with the formation of HRE-binding protein, like HIF-1α. After 12 h hypoxia induction, the amount of DNA–protein complex increased significantly comparing with the normoxic group (Fig. 7D lane 2 & 4), but was apparently inhibited by K5 treatment (Fig. 7D lane 5). K5 had little effect in blocking the formation of the protein–DNA complex under normoxia condition (Fig. 7D lane3).

Taken together, these results suggested that K5 decreased HIF-1α protein level and impaired nuclear HIF-1α accumulation, indicating that HIF-1α maybe a candidate targeting molecule of K5.

## Discussion

In our previous studies, we observed the endothelial cell-specific inhibition and anti-angiogenic activity of K5 *in vitro* and in some animal models such as oxygen-induced retinopathy (OIR), diabetes and alkali-burn-induced corneal neovascularization [7], [9]–[12], [22]. To further elucidate the effect of K5 on cancer metastasis, we established a spontaneously metastatic mouse model of LLC and analyzed the cellular changes and signal molecule alterations in LLC cells with K5 treatment. Our results showed for the first time that K5 inhibits both tumor growth and metastasis in LLC via the dual effects of anti-angiogenesis and suppression of tumor cell motility by targeting HIF-1α pathway. K5 decreased HIF-1α protein level and impaired nuclear HIF-1α accumulation. Consequently, K5 down-regulated the down-stream genes expression of CXCR4 and VEGF, which may be responsible for the dual inhibitory effects of K5 on angiogenesis and tumor metastasis.

The antiangiogenic effect was proved by CD34 immunostaining with marked decrease of MVD in LLC tumor tissues treated with K5 (Fig. 3A). This result is similar to our previous observation in K5-treated human hepatocellular xenografts tissues [8], and also consistent with the report of Jin and Jiang that K5 combined with irradiation or the modified K5 hold an enhanced antitumor activity in a mouse tumor model of lung cancer [13], [29]. VEGF is the most potent and specific growth factor enhancing tumor microvessel density and inducing angiogenesis. The previous studies showed that K5 treatment resulted in dose-dependently reduction of VEGF expression and release in endothelial cells [22], [29], [30]. In this study, we demonstrated that K5 reduced production of VEGF in tumor cells *in vitro* and *in vivo*. However, K5 did not hold a significant effect on cell proliferation and apoptosis of LLC *in vitro* as shown in figure 4A and B. These results suggested that the inhibitory effect of K5 on LLC growth and metastasis may be mediated by anti-angiogenesis through down-regulation of VEGF.

Although K5 had no effect on cell proliferation and apoptosis of LLC, here, we reported a striking direct blocking of K5 on the chemotaxis movement of LLC cells. K5 has been consistently characterized as an endothelial cell-specific inhibitor [6], [15] and carries out its inhibitory effects by binding to some special sites such as voltage-dependent anion channel [31] and glucose-regulated protein 78 presented on endothelial cells [32], [33]. It was shown previously that K5 significantly inhibited the migration of endothelial cells induced by VEGF and the aggregation of inflammatory cells in the process of angiogenesis [9], [15], [16]. Moreover, K5-engineered glioma cells blocked the migration of tumor-associated macrophages and suppressed cancer-associated angiogenesis [14]. In the current study, we demonstrated that K5 exhibited a concentration-dependent inhibitory effect on the chemotaxis movement of LLC cells induced by SDF-1α (Fig. 4D), which is a small chemoattractant cytokine and the high-affinity ligand of CXCR4 [34]. SDF-1α is constitutively secreted by fibroblasts in several different organs/tissues including bone marrow, lymph node, lung and liver which are the most common target sites for distant metastasis [35]. SDF-1α and its specific receptor CXCR4 expressed on the membrane of tumor cells form the SDF-1α/CXCR4 chemokine axis, which plays a pivotal role in migration, invasion and metastasis of some malignant tumors including LLC [27]. Here, we observed CXCR4 expression on the membrane of LLC cells and SDF-1α dose-dependently inducing migration of LLC cells (Fig. 4C & Fig. 5B). It demonstrated that the SDF-1α/CXCR4 axis and chemotaxis movement were involved in the metastasis of LLC cells. We also showed that K5 treatment markedly down-regulated CXCR4 expression both in LLC cells and LLC-grafted tumor tissues (Fig. 5A, B & C). These suggested that the anti-metastatic effect of K5 may at least in part come from the blocking of SDF-1α/CXCR4 chemotaxis movement of LLC cells.

As mentioned above, both VEGF and CXCR4 can be down-regulated by K5 in LLC cells. Actually, CXCR4 and VEGF are the collaborators in tumor metastasis. Cross-talking between SDF-1α/CXCR4 axis and VEGF pathway is proved to favor tumor progression as angiogenesis and migration are requisites to metastasis promotion [36], [37]. VEGFR1 positive haematopoietic progenitor cells initiate the pre-metastatic niche in metastatic target organ such as lung and induce SDF-1α expression in pulmonary fibroblasts by integrin α_4_β_1_/fibronectin signal pathway [27]. SDF-1α in pre-metastatic niche may capture circulating CXCR4^+^ cancer cells and then provide one pathway for metastasis formation [27], [38]. Our results showed that SDF-1α was significantly up-regulated in metastatic lung tissues of LLC-bearing mice compared with the normal lung tissues. And K5 can significantly down-regulate SDF-1α expression in metastatic lung tissues of LLC (Fig. 5D). These results suggested that K5 may also alter the pre-metastatic niche by regulating the cross-talking between SDF-1α/CXCR4 and VEGF. The further studies are needed to investigate the exact mechanisms.

Adaptation to hypoxic conditions is a critical step in tumor progression and is, in part, regulated by HIF-1α [39]. HIF-1α translocates into the nucleus where it binds to HRE on genes induced by hypoxia and activates transcription of downstream genes, including VEGF and CXCR4 [20], [21], [40]. Here we confirmed that hypoxia induced the gene expression of VEGF and CXCR4 and enhanced the protein level of intracellular HIF-1α in LLC cells as shown in figure 3C, figure 5A, B and figure 6C. Moreover,we used short hairpin RNA (shRNA) to silence the expression of HIF-1α in LLC cells,and then observed the corresponding reduction of CXCR4 and VEGF (Fig. 6A). We also observed that hypoxia enhanced the SDF-1α-induced chemotaxis movement of LLC cells and shRNA of HIF-1α blocked it (Fig. 6B). These data suggested that the gene expressions of CXCR4 and VEGF in LLC cells mainly depend on the level of HIF-1α under hypoxia.

**Figure 8:**
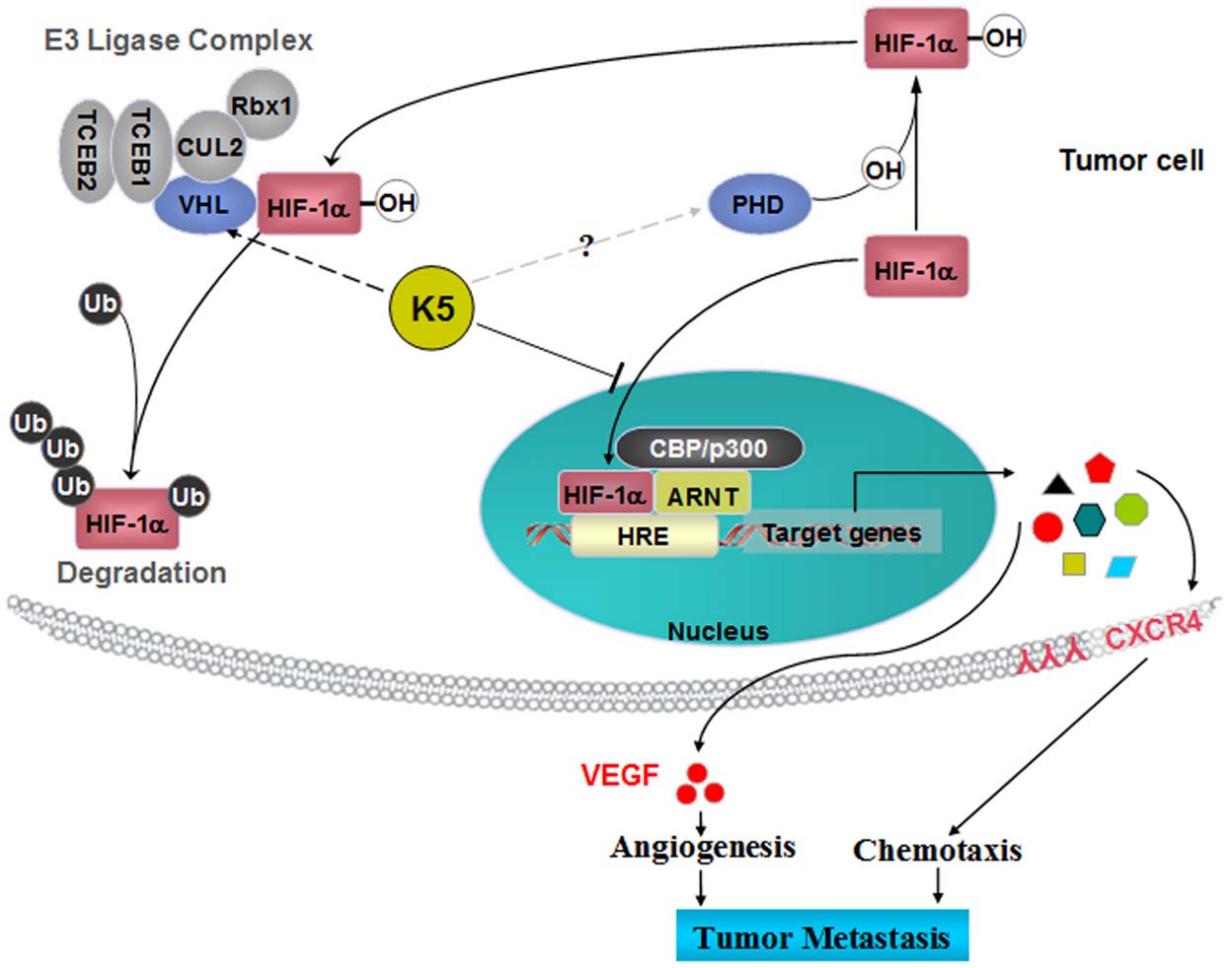
A schematic overview of the potential mechanism involved in K5-mediated inhibition of HIF-1α in tumor cells. K5 up-regulates VHL and consequently promotes ubiquitin-proteasome medicated protein degradation of HIF-1α. Moreover, K5 decreased HIF-1α protein stabilization, reduced nuclear HIF-1α accumulation and then inhibited transcriptional activation. Consequently, K5 down-regulated the gene expression of CXCR4 and VEGF, which were the downstream genes of HIF-1α pathway. VEGF and CXCR4 play key roles in angiogenesis and chemotaxis migration which both are requisites to metastasis promotion. This may be responsible for the dual inhibitory effects of K5 on tumor metastasis.

HIF-1α pathway has been proposed as a suitable target for future anticancer therapy [41]–[43]. Our previous studies confirmed that K5 reduced the HIF-1α levels in the retina of retinopathy model and the retinal capillary endothelial cells [22]. The present study demonstrated that HIF-1α was expressed apparently both in nuclear and cytoplasmic compartments of LLC cells induced by hypoxic conditions, and K5 significantly down-regulated HIF-1α expression *in vivo* and *in vitro* (Fig. 6C & D). The protein level of intracellular HIF-1α is determined mainly by its rate of proteasomal degradation [44]. Briefly, HIF-1α is hydroxylated by prolyl hydroxylases (PHDs) under normoxic conditions. This modification allows the binding of the tumor-suppressor protein von Hippel-Lindau (VHL) to HIF-1α, and then promotes the formation of E3 ubiquitin ligase complex. The VHL protein mediates polyubiquitination of the HIF-1α subunit at three lysine residues, which results in its degradation by the proteasome. Thus, PHDs and VHL may be the candidate target molecules for the stabilization of HIF-1α during hypoxia (Fig. 8). It has been confirmed that K5 promoted the ubiquitin-proteasomal degradation of HIF-1α by inducing VHL, resulting in the decreased protein level of intracellular HIF-1α (data no shown). In the current studies, K5 treatment significantly reduced the amount of HIF-1α in cytoplasm and lead to a more marked reduction of HIF-1α in nucleus, suggesting that K5 not only down-regulated the protein level of HIF-1α, but also inhibited HIF-1α nuclear accumulation (Fig. 7A, B & C). The rapid nuclear translocation of HIF-1α represents an efficient way to escape from degradation and is the essential steps for HIF-1α in the transactivation of hypoxia-responsive genes [45]. The anti-metastasis effect of K5 is likely to be mediated by suppressing the protein stabilization and nuclear accumulation of HIF-1α, consequently inhibited the HIF-1α transcriptional activity that could be responsible for decreasing gene expression of VEGF and CXCR4, resulting in the inhibition of angiogenesis and tumor chemotaxis movement which are indispensable steps in the progression of metastasis (Fig. 8).

In conclusion, previous studies have shown that HIF-1α pathway played important roles in the tumor growth and metastasis including major types of human tumors such as lung cancer, gastric cancer, breast cancer, prostate cancer, pancreatic cancer etc [17], [43], [46], [47]. Therefore, HIF-1α-targetting therapies provide new insights into the treatment of cancer [3], [39], [41], [42]. The present study demonstrated that K5 can inhibit LLC tumor growth and metastasis by regulating HIF-1α and its downstream genes of VEGF and CXCR4. Moreover, the similar inhibitory effects of K5 on human lung cancer cell line A549 were also observed (Supplementary Fig. S1). Our finding implies the potential role of K5 as a promising HIF-1α-targeting molecule in the treatment of tumor growth and metastasis.

## Supporting Information

Figure S1 **K5 has similar effects on A549 human lung cancer cells as LLC.** (A) K5 dose-dependently down-regulated CXCR4 expression in A549 cells treated with hypoxia. CXCR4 protein levels in cell lysates were measured by Western blot analysis, semi-quantified by densitometry and normalized by β-actin concentration. (B) A549 cells were serum-starved and exposed to either normoxia or hypoxia and the chemotaxis movement induced by SDF-1α (50 ng/ml) were examined in a modified Boyden Chamber assay with the presence of K5 at different concentrations. The migrated cells were observed and quantified by photographing with HE stain. K5 remarkably inhibits the cell migration in a dose dependent manner. Data are presented as Mean ± SD, n = 3,^ #^ *P*<0.05,^ *^ *P*<0.01 *vs* control (group2), as a percentage of inhibition.
(TIF)
