# Supplementary figures and images for: Dual Inhibition of Plasminogen Kringle 5 on Angiogenesis and Chemotaxis Suppresses Tumor Metastasis by Targeting HIF-1α Pathway
Source: PLoS One. 2012 Dec 31;7(12):e53152. doi: 10.1371/journal.pone.0053152 (PMC3534244; doi:10.1371/journal.pone.0053152)

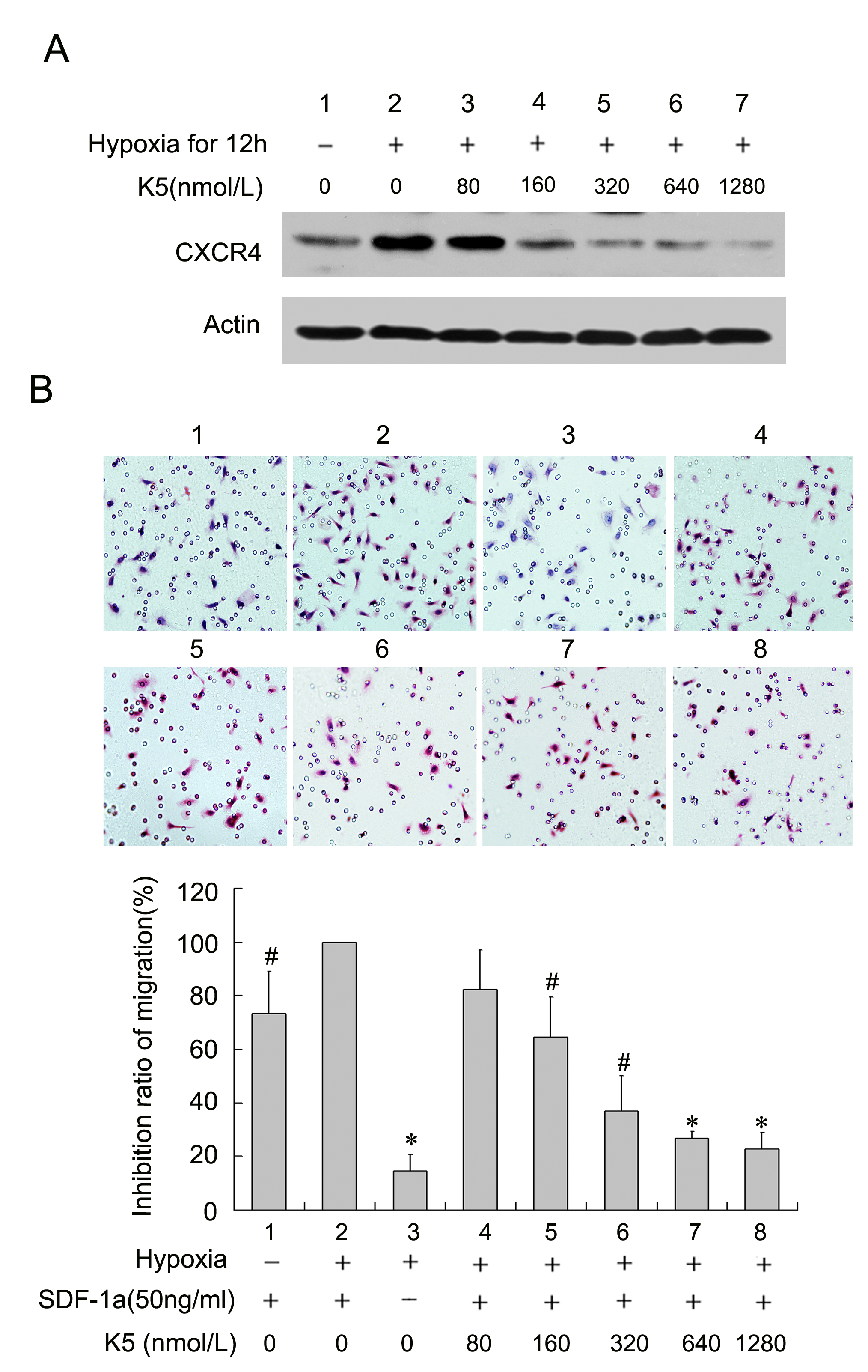

Supplement: Figure S1 — K5 has similar effects on A549 human lung cancer cells as LLC. (A) K5 dose-dependently down-regulated CXCR4 expression in A549 cells treated with hypoxia. CXCR4 protein levels in cell lysates were measured by Western blot analysis, semi-quantified by densitometry and normalized by β-actin concentration. (B) A549 cells were serum-starved and exposed to either normoxia or hypoxia and the chemotaxis movement induced by SDF-1α (50 ng/ml) were examined in a modified Boyden Chamber assay with the presence of K5 at different concentrations. The migrated cells were observed and quantified by photographing with HE stain. K5 remarkably inhibits the cell migration in a dose dependent manner. Data are presented as Mean ± SD, n = 3, # P<0.05, * P<0.01 vs control (group2), as a percentage of inhibition. (TIF) [file pone.0053152.s001.tif]
